# Hazard Mapping of Subterranean Termite Attacks in Makassar City, South Sulawesi, Indonesia

**DOI:** 10.3390/insects11010031

**Published:** 2019-12-31

**Authors:** Astuti Arif, Giselawati Putri, Musrizal Muin

**Affiliations:** Forestry Department, Faculty of Forestry, Hasanuddin University, Makassar 90245, Indonesia; astutiarif73@gmail.com (A.A.); musrizal@yahoo.com (M.M.)

**Keywords:** south sulawesi, *Schedorhinotermes* sp., *Coptotermes gestroi*, *Microcerotermes serrula*

## Abstract

Termites are distributed throughout the world and often cause economic losses. This study aims to; (1) analyze the relationship between the distribution of termite species and the environmental conditions of Makassar city; (2) determine the level of attack in the Makassar City; and (3) map the risk of termite attack in the Makassar City. Samples of *Pinus merkusii* (2 × 2 × 20 cm) were placed at 13 observation stations and covered using polyvinyl chloride (PVC) tubes (4″ in diameter, 25-cm in length). Samples remained in place for 6 months, at which point the presence of termite attacks as well as their intensity and frequency were analyzed. Three species of termites were found among the 13 stations: *Schedorhinotermes* sp., *Coptotermes gestroi*, and *Microcerotermes* serrula. Their presence was significantly influenced by environmental factors at each station. The study results showed a low rate of attack in several subdistricts of Bontoala, Biring Kanaya, Makassar, Mamajang, Mariso, Manggala, Panakukang, Rappocini, Tallo, Tamalanrea, Tamalate, Ujung Pandang, Ujung Tanah, and Wajo districts. A medium level of attacks was found in subdistricts within Biring Kanaya, Manggala, Mamajang, Panakukang, Rappocini, and Tallo Districts. Finally, a high risk of termite attacks was present in several subdistricts of Biringkanaya, Tamalantera, Rappocini, Manggala, and Tamalate districts.

## 1. Introduction

Termites are arthropod organisms in the tropics and subtropics [1] and in warm temperate regions [2]. They play a crucial role in ecosystems by modifying the chemical, physical, and biological properties of soil [3,4]. They can recycle living, dead, and decaying plant matter through decomposition activities [5], as well as restore nutrients in soils and create fertile areas on land that is arid or otherwise poor quality [6]. Termites can also bio-convert waste paper to generate valuable biogenic products that enhance soil properties [7]. Their activity in soil has been found to increase water infiltration rates, as exemplified by foraging galleries of subterranean termites [8,9]. Because of their influence on the distribution of natural resources, such as water and nutrients in the landscape, termites also affect the diversity of soil microbes, plants, and animals [10].

Although, termites are ecologically crucial, several species are also significant pests, both in natural forest habitats and in urban areas in particular. Among the 3106 species identified [11], approximately 28 species are considered invasive and have dispersed beyond their native territory [2,12]. Termite-related damage to structural components made of wood in buildings is a severe problem in tropical and subtropical countries [5], causing substantial economic losses. The amount of such losses varies by country as well as by termites species. In the United States, for example, eastern subterranean termites (*Reticulitermes flavipes*) cause approximately $2 billion in damage annually [13]. In Indonesia, losses approach 2.8 billion rupiahs annually [14]. Damage caused by termites in agroforestry habitats in South Sulawesi Province has been reported by Arif et al [15], and Arif and Nurdianti [16] have studied termite-related damage of government buildings in the same area. However, there is generally a lack of information related to the economic losses associated with termites in South Sulawesi.

Makassar is the capital of South Sulawesi Province, located on Celebes Island, Indonesia. The island is geo-ecologically located within the Wallace Area, which has characteristic flora and fauna that are distinct from those in continental Asia and Australia. Approximately 42% of the total land area of Makassar (175.77 km^2^) in the yard and land of office/residential buildings [17]. The land use of the city of Makassar has changed in 2019, in line with the increase in population (1.29% in population growth) and subsequent housing needs. Such extensive environmental changes undoubtedly affect the feeding behavior of local organisms, including termites. Termite attacks in the city have been reported by Arif et al. [18], who studied the wooden components of the Karaeng Pattingalloang Museum, Somba Opu Fort, as well as by Nurhamila [19], who found evidence of termites feeding on ancient manuscripts at the La Galigo Museum. Although more extensive information on the incidence of termite attacks in the city is lacking, the risk of termite attacks likely occurs throughout the Makassar area. In general, people do not consider termite prevention prior to construction and may use materials susceptible to termite attack, including untreated wood from very vulnerable (III and IV) group. In this study, we aimed to analyze the effect of environmental factors (soil and climate) on the incidence, intensity, and frequency of subterranean termite attacks. We also aimed to produce maps based on the risk of subterranean termite attacks in Makassar City, South Sulawesi, Indonesia. 

## 2. Material and Methods

### 2.1. Study Sites

**Figure 1:**
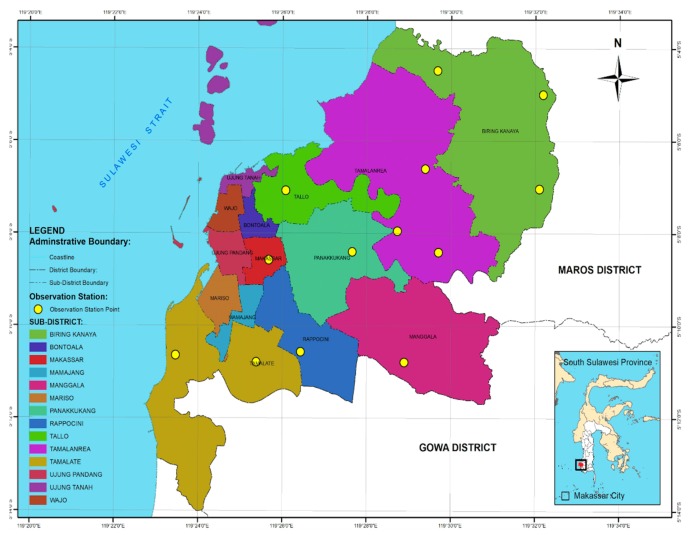
Map of observation stations in Makassar City, South Sulawesi Province, Indonesia.

This study was conducted in March–September 2019, in Makassar City (5°8′ to 5°6′ S, 119°24′ to 119°17′ E). Elevation ranged from 1 to 25 m above sea level. Thirteen observation stations were determined by placing a grid over the Makassar city administration map as shown in Figure 1. Two PVC tubes (4′ in diameter, 25 cm in length) were vertically placed at each station to a depth of half the tube length. Four stakes of pine wood (*Pinus merkusii*), each measuring 2 × 2 × 20 cm, were used for each tube. A total of 104 stakes were prepared. Stakes were left in place for two observation periods (3 and 6 months). All stakes from one tube were observed for the occurrence of termite soil attacks at the third month and all stake of another tube were observed at the end on observation. 

### 2.2. Collection of Termites and Determination of Species

Termite specimens from each pine stake were placed in a collection vial containing 70% ethanol. The morphological characteristics of the soldiers were observed using Stemi 2000 stereo microscopes with phototube cameras, and the termites were identified by species based on matching with the determination key [20,21,22,23].

### 2.3. Collection, Preparation, and Analysis of Soil Samples

Soil samples were collected from excavations when observation stations were placed. Soil samples were stored in the Laboratory of Silviculture and Plant Physiology and air-dried. The soil characteristics measured were pH, C-organic content, and texture (percentages of sand, silt, and clay and the sand/clay ratio). Soil pH was measured using a pH meter, the soil C-organic content was determined using the Walkley-Black method [24], and the soil texture was determined using the hydrometer method [25].

### 2.4. Measurement of Climatic Factors

Climatic factors observed in the study were temperature and relative humidity. Temperature and relative humidity were measured using a thermo-hygrometer placed near the observation station. These data were just before collecting the termite specimens in each observation period. 

### 2.5. Analysis of Environmental Factors on the Incidence of Termite Attacks 

The effects of the environmental factors (i.e., soil characteristics and climatic factors) on the incidence of termite attack on the pine stakes were analyzed using binary logistic regression analysis [26]. Attack by subterranean termites was allocated a binomial categorical variable equal to 1, if termites were present, or 0, if they were absent. The environmental characteristics were considered continuous independent variables.

### 2.6. Analysis of the Effect of Environmental Factors on the Damage Intensity 

The intensity of wood damage is expressed as the percentage of loss from the cross-sectional area due to termite attack according to ASTM D 1758-06 [27], modified for the size of the samples used in this study (2 × 2 × 20 cm). The damage intensity was rated according to the seven classes listed in Table 1. The highest intensity of termite-related damage is described as severely damaged wood (score 0), while the lowest intensity is rated as minor damage (score 10).

The effects of the environmental factors were analyzed using ordinal logistic regression analysis. An ordinal categorical variable was used for the intensity score. The environmental characteristics were considered continuous independent variables.

### 2.7. Analysis of the Effect of Environmental Factors on the Frequency of Soil Termites in Baits

The frequency of attacks is expressed as the ratio between the amount of each sample attacked by termites and the depth to which the stake was buried. The frequency data fall into six classes according to Cookson and Trajstman [28], as presented in Table 2.

Poisson logistic regression analysis was performed to determine the effects of the environmental factors on the frequency of termite attack, following the prediction model of Arinana et al [26].

### 2.8. Risk of Termite Attack 

The combination of the frequency of termite attack and the intensity of wood damage indicates the risk level of termite attack at the study site. The frequency and intensity are directly proportional to the risk level. The high frequency, as well as the high intensity, were associated with a high level of risk. Three risk classes were defined by classifying 36 combinations of attack frequency and intensity of damage to wood (Table 3), and these combinations provide the best means to estimate the risk class of termite attack.

## 3. Results and Discussion

### 3.1. Environmental Conditions 

*a. Soil pH*

The soil pH at the observation stations ranged between 6.03 and 8.84 (average, 7.79 ± 0.91). The soil pH at the three locations with termite activity was 6.03, 8.28, and 8.84. This finding indicates that soil pH is not a limiting factor in the activity of subterranean termite attacks. The activity of termites can increase the pH of soil, which can cause the content of nitrogen and phosphorus, as well as the concentrations of copper, molybdenum, and manganese, to be relatively higher than in unmodified soils [29]. This outcome is in line with research by [30], who found that *Cubitermes fungifaber* can increase the pH in soils that have a low initial pH value; increase organic carbon, water content, and the amount of kaolinite; and reduce quartz. The main effect of termite activity is the ability to improve soil quality (local microsite). Therefore, termites are potentially an essential source of heterogenesis in soil systems in tropical forests.

*b. C-organic content*

The organic matter content of soil is one of the limiting factors for soil fertility whose function is to increase nutrients and as a nutrient buffer. The C-organic content varied from 0.82 to 1.60% (1.16 ± 0.24%). This value was categorized as being very low to very high [31]. Organic matter is a source of energy for the macro and micro-fauna of the soil. The addition of organic matter in the soil increases microbiological activity and population, especially for organisms related to the decomposition and mineralization of organic matter [32]. In addition, the C-organic content of the soil provides benefits to surrounding soil microorganisms to meet their daily needs [33]. The quality and availability of food sources in the environment determine the composition and size of the termite community. Termite activity increases with the application of various mulches as nutrient resources on soil surfaces [34]. Soil termites can also play a role in distributing organic matter into the deeper layers of soil and can help in the process of mixing organic matter and soil [35].

*c. Soil Texture*

The soil texture was determined by the ratio of the sand, silt, and clay fractions based on the soil texture triangle [36]. We found six types of soil textures at the experimental stations, namely clay (stations S1, S2, S5, and S9), sand (S11), silty clay (S13), clay (S6 and S7), sandy clay (S3, S4, S8, and S10), and clay (S12). Locations that had termite attack activity had clay texture, namely clay (S6), sandy clay (S3), and clay (S12). Clay can store nutrients better than other textures [37], which is explained by it high organic matter content. Clay has a balanced composition between coarse and fine fractions, and it is often considered to be the optimal texture, especially for agriculture. Clay can increase the organic matter content of the soil, leading termites to build nests [38]. In general, clay absorb nutrients better than sand, while sand improves drainage, aeration, and ease of processing [36]. 

*d. Temperature and Humidity*

Temperature and humidity affect the survival, growth, development, and survival of termites. Temperature strongly influences feeding activities [39], and according to Prasetiyo and Yusuf [40], both temperature and humidity affect the activity and behavior of termites. Termites’ preferred time for foraging is from midday to early afternoon; however, they also prefer shade, which helps to create the optimal temperature. The temperature recorded in this study ranged from 30.13 to 33.10 °C (31.23 ± 0.98 °C), which was conducive for subterranean termite activity. Nandika et al [41] reported that the optimum temperature for termites is between 15 and 38 °C. Also, Arinana et al [26] found active termites, including *Coptotermes curvignathus*, *Schedorhinotermes javanicus*, *Microcerotermes insperatus*, and *Macrotermes gilvus*, in a residential area in Jakarta, where the average temperature ranged from 29.3 to 30.1 °C (min. 21.9–26.1 °C and max. 35.3–38.8 °C). In addition, Fei and Henderson [42] found that the feeding activity (consumption rate) of *C. formosanus* increased as temperature became warmer (30–33 °C).

Humidity indicates the amount of water contained in air. The humidity at the 13 stations ranged from 51.00% to 75.33% (62.05% ± 7.18%). These values were below the optimum humidity range of termites reported by Nandika et al [41], which was between 75 and 90%. However, the values obtained in the current study were not very different from those found by Arinana et al. [26]. In that study, the average humidity varied from 68.2 to 72.3%, with a minimum value of 35.4% during the day and a maximum value of 91.9% in day-spring. Moisture plays an important role in the life of termites because they require high humidity to prevent dehydration. Unstable soil temperature and humidity make soil micro-fauna move from an area to seek places with more stable conditions. Subterranean termites typically avoid open areas and gravitate toward areas with more stable habitat conditions [43].

### 3.2. Termite Distribution

Three genera of subterranean termite were identified in the current study, based on morphological and morphometric characters, namely *Schedorhinotermes* and *Coptotermes* (family: Rhinotermitidae) and *Microcerotermes* (family: Termitidae).

*a. Schedorhinotermes* sp.

**Figure 2:**
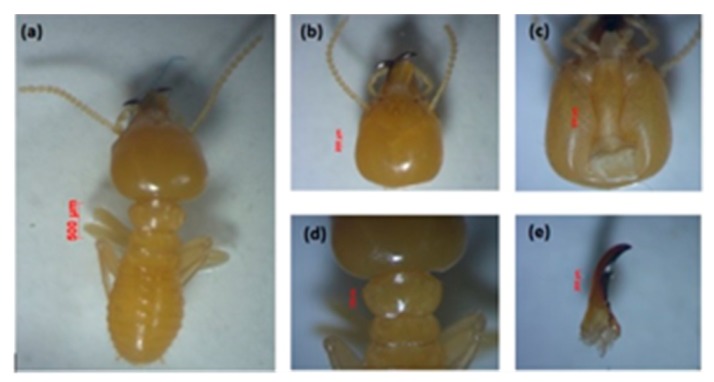
Major soldier of *Schedorhinotermes* sp.: (**a**) body shape, (**b**) part of the head with antennas, (**c**) postmentum, (**d**) pronotum, and (**e**) left mandible.

**Figure 3:**
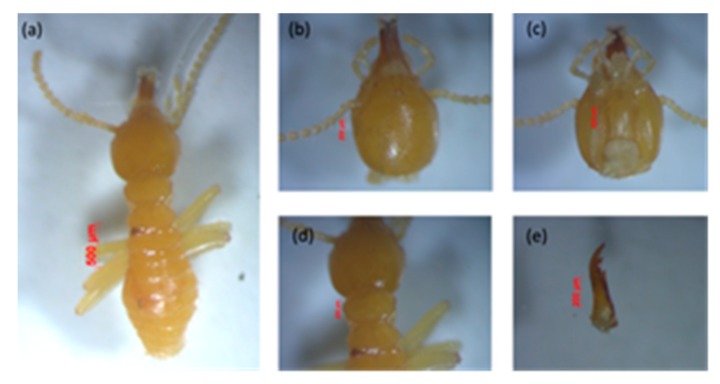
Minor soldier of *Schedorhinotermes* sp.: (**a**) body shape, (**b**) part of the head with antennas, (**c**) postmentum, (**d**) pronotum, and (**e**) left mandible.

This species is easily recognized because of their dimorphic soldiers—a major soldier is shown in Figure 2, and minor soldier in Figure 3. This species is characterized by the shape of the labrum, resembling an elongated hourglass with a thin bilobed anterior edge, and mandible tip extending and slightly curved [44]. The head capsule of the major soldier is round and yellowish red in color, and it has thick, serrated mandibles. *Measurement of five major soldiers*: The head length without mandible (HL) was 1.39–1.52 mm, the maximum width of the head (mHW) was 1.40–1.44 mm, the head width at the mandible base (bHW) was 0.64–0.84 mm, and the left mandible length (LML) was 0.87–1.00 mm. The postmentum length was 0.88–1.07 mm, the maximum width of the postmentum was 0.42–0.43 mm, and the minimum width of the postmentum was 0.18–0.29 mm. The pronotum length was 0.80–0.85 mm and the maximum width of the pronotum was 0.34–0.51 mm, and the number of antennas was 16 segments. The value of the bHW/LML index was 0.44–0.58, the mHW/HL index was 0.95–1.02, and the LML/HL index was 0.61–0.73.

*Measurement of five minor soldiers*: The minor soldiers were characterized by a labrum extending beyond the tip of the mandibles. They had an oval-shaped head capsule, and the head length without mandible (HL) was 0.80–0.97 mm, the maximum width of the head (mHW) was 0.71–0.78 mm, the head width at the mandible base (bHW) was 0.35–0.42 mm, and the left mandible length (LML) was 0.18–0.19 mm. The postmentum length was 0.51–0.53 mm, the maximum width of the postmentum was 0.39–0.21 mm, and the minimum width of the postmentum was 0.15–0.18 mm. The pronotum length was 0.22–0.39 mm, and the maximum width of the pronotum was 0.34–0.51 mm, and the number of antennas was 15–16 segments. The value of the bHW/LML index was 0.46–0.56, the mHW/HL index was 0.74–0.94, and the LML/HL index was 0.60–0.82.

The presence of dimorphic soldier characterizes the genus *Schedorhinotermes* as cited by Arumugam et al. [45]. The species is generally yellowish in color, and it emits a strong and unpleasant odor.

*b. Coptotermes gestroi*

**Figure 4:**
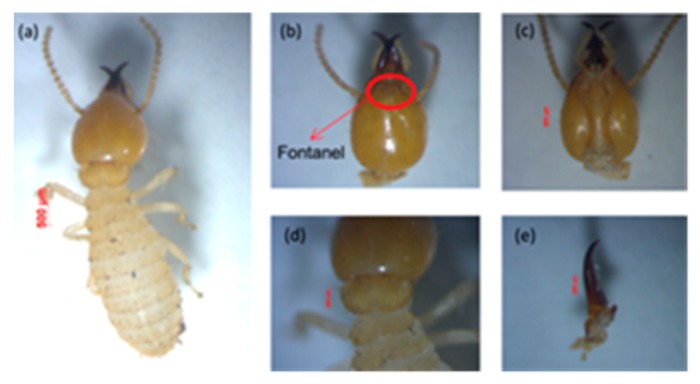
View of *Coptotermes* sp.: (**a**) body shape of soldier, (**b**) head with antenna and fontanelle, (**c**) postmentum, (**d**) pronotum, and (**e**) left mandible.

This species was easily identified because of the wide opening in the fontanel of the head, which looks very clear with light yellow in color [46]. It generally secretes a milky white liquid, as can be seen in Figure 4, that is used for self-defense against enemies. In addition, the head is characterized by long and sparse hair. 

*Measurement of five soldiers:* They had an oval-shaped head capsule, and the head length without mandible (HL) was 1.33–1.51 mm, the maximum width of the head (mHW) was 1.10–1.14 mm, the head width at the mandible base (bHW) was 0.49–0.75 mm, and the left mandible length (LML) was 0.66–0.72 mm. The postmentum length was 0.85–0.97 mm, the maximum width of the postmentum was 0.39–0.45 mm, and the minimum width of the postmentum was 0.19–0.39 mm. The pronotum length was 0.76–0.86 mm, and the maximum width of the pronotum was 0.35–0.42 mm. The head and pronotum had sparse hair, while the postmentum had several long hairs. The head was narrow anteriorly, with a bHW/mHW index of 0.44–0.64; head width nearly long head, with an mHW/HL index of 0.77–0.83; yellow to orange in head color. Mandibles curved in weakly, with curvature beginning at the apical third of mandible length; mandibles exceeded half the head length, with an LML/HL index of 0.64–0.68. Antennas had 15 segments and the narrowest part of the postmentum was located about half of the distance from the posterior margin and the widest point. Based on the determination key of *Coptotermes* species [23], the morphological characteristics of the observed subterranean termites were close to *C. gestroi*.

*c. Microcerotermes serrula*

**Figure 5:**
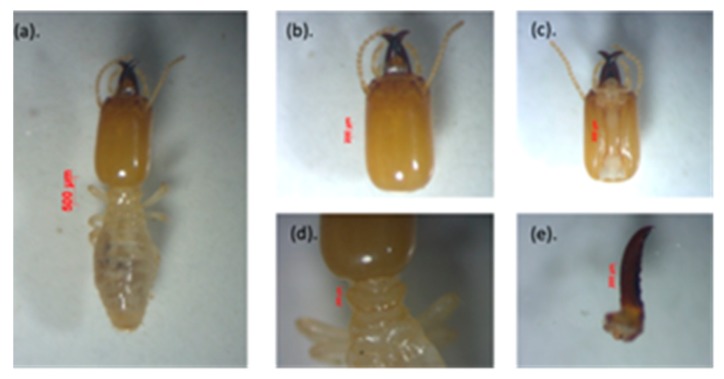
View of *Microcerotermes serrula*: (**a**) individual body shape of the soldier, (**b**) head with antennas, (**c**) postmentum, (**d**) pronotum, and (**e**) left mandible.

This species is easily recognized because of the rectangular shape of the head capsule, which is a light brown, and the serrated mandibles, as shown in Figure 5. *Measurement of five soldiers*: The soldiers had a rectangular head capsule, the head length without mandible (HL) was 1.64–1.73 mm, the maximum width of the head (mHW) was 0.40–0.97 mm, the head width at the mandible base (bHW) was 0.35–0.42 mm, and the left mandible length (LML) was 0.81–0.91 mm. The postmentum length was 0.85–1.05 mm, the maximum width of the postmentum was 0.32–0.35 mm, the minimum width of the postmentum was 0.14–0.94 mm; the pronotum length was 0.59–0.63 mm, and the maximum width of the pronotum was 0.30–0.34 mm. Antennas had 12 segments. The value of the bHW/LML index was 0.38–0.44, the mHW/HL index was 00.23–0.58, and the LML/HL index was 0.47–0.54. The morphological and morphometric characteristics of the specimens are identical to the *M. serrula* species found by Nurhadi [47].

### 3.3. Attack Rate of Subterranean Termites

**Figure 6:**
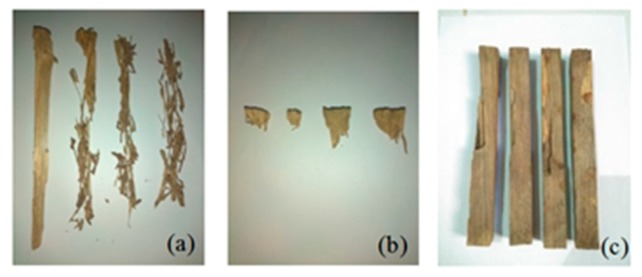
Condition of stakes after being attacked by subterranean termites (**a**) *Coptotermes gestroi*, (**b**) *Schedorhinotermes* sp., and (**c**) *Microcerotermes serrula*.

The condition of the stakes after six months of testing shows the variation of damage depending on the species that attacked, as shown in Figure 6. Only eight samples from 104 were attacked by *Coptotermes gestroi* after testing. Figure 6a shows the condition of these stakes. The intensity of termite attack, based on the visual rating, was zero for all samples, which means that more than 75% of the wood was removed by subterranean termites. The damage caused by *C. gestroi* was very severe, and the wood residue is in the form of long and thin flakes. White spots were found on the surface of the sample, which was likely white liquid secreted by soldier termites.

Only eight of the 104 stakes were attacked by *Schedorhinotermes* sp. after testing for 6 months. The intensity of termite attack, based on the visual rating, was zero for all samples, which means that more than 75% of the wood was removed by subterranean termites. Characteristics of attack by *Schedorhinotermes* sp. can be seen from the stakes shown in Figure 6b. The damage was very severe, and the stakes were almost entirely consumed; only the edges that were labelled with the sample code was not consumed. Figure 6c shows that the damage due to *M. serrula* attack was not as severe as that caused by the other two species. The intensity of termite attack based on the visual rate was six for all samples, which means that more than 30% of the wood was removed by subterranean termite. The characteristics of the damage caused by *M. serrula* showed that the termites attacked the surface of the sample to form a tunnel. In addition, there was a change in color on the surface of the samples.

### 3.4. Termite Attack Analysis and Risk Mapping

a. Effect of Environmental Factors on the Incidence of Subterranean Termite Attack

Evidence of termite attacks was only found at 3 of the 13 observation stations (S3, S6, and S12). The effects of the environmental factors, consisting of soil characteristics (soil pH, C-organic content, sand, silt, clay, sand/clay ratio) and climate (temperature and humidity), on the incidence of subterranean termite attack on the samples were analyzed using binary logistic regression analysis. The results showed that the deviation value (*p* = 1.00) and Pearson’s chi-square test (*p* = 1.00) were greater than α = 0.05, which means that there is not enough evidence to say that the resulting model does not match the data. In other words, the two tests indicate that the resulting model fits the data. The results of simultaneous tests or Omnibus tests indicate that the p-value (0.00) is less than α = 0.05, which means that there is at least one variable among the environmental factors that significantly affects the intensity of termite attack at a 95% confidence level. The partial estimator test or the Wald chi-square test showed that all environmental factor variables had a p-value less than 0.05, which means that all environmental factor variables significantly affected the incidence of subterranean termite attack (*Schedorhinotermes* sp, *Coptotermes gestroi*, *Microcerotermes serrula*) at a 95% confidence level. This finding is contrast with the research conducted by Arinana et al [26] showing that soil and climate factors do not affect the presence of termites of *C. curvignathus*, while temperature factors affect *Microtermes insperathus* and *Macrotermes gilvus* are influenced by C-organic content.

b. Effect of Environmental Factors on the Intensity of Subterranean Termite Attack

The intensity of the termite attack varied between stations. Two stations had an attack intensity with a value of zero (high), and one station had an attack intensity rated as six (medium). The other stations had an attack intensity of 10 (low), although caution would still be needed against the possibility of a termite attack. The effects of the environmental factors on the intensity of wood damage due to subterranean termite attack were analyzed using ordinal logistic regression analysis. The results showed that the deviation value (*p*-value = 1.00) and Pearson’s chi-square test (*p*-value = 1.00) were greater than α = 0.05, which means that there is not enough evidence to say that the resulting model does not match the data. In other words, the two tests indicate that the resulting model fits the data. The results of simultaneous tests or Omnibus tests indicate that the *p*-value (0.00) is less than α = 0.05, which means that at least one of the environmental factors significantly affects the intensity of termite soil attack at a 95% confidence level. The partial parameter estimation test or the Wald chi-square test shows that all environmental factors have *p* values greater than 0.05, which means that none of them have a significant effect on the intensity of the termite attack at a 95% confidence level. 

Each termite species consumed a variety of wood samples during the 6 months of baiting. The monthly average wood consumption by *Schedorhinotermes* sp, *C. gestroi*, and *M. serrula* were 7.48 g, 6.64 g, and 1.47 g, respectively. These findings were much lower than those reported for *Coptotermes travians* (478.2–643.7 g/month) [48]. The factors that affect feeding activity of termites are environmental conditions [26], foraging distance, foraging territory, and colony size [48].

c. Effect of Environmental Factors on the Frequency of Termite Attack

Termite attack frequency data in the complete sample of each bait station is not attacked with a frequency of 0% (no attack), while the other three stations that had stakes attacked by termites demonstrated a frequency between 30% (attack moderate) to >40% (very high attack) based on [28]. The effects of the environmental factors on the frequency of wood damage due to subterranean termite attack were analyzed using Poisson logistic regression analysis. The results showed that the deviation value (*p* = 0.999) and Pearson’s chi-square test (*p* = 1.00) were higher than α = 0.05, which means that there was not enough evidence to say that the resulting model does not agree with the data. In other words, the results of both tests suggest that the resulting model fits the data. Based on simultaneous tests or Omnibus tests, the *p*-value (0.00) was less than α = 0.05, which means that at a 95% confidence level, there was at least one environmental factor that significantly affected the frequency of termite attacks. The partial estimator test or the chi-square test of Wald showed that all environmental factors had *p*-values higher than 0.05, which means that none of the environmental factors had a significant effect on the frequency of termite attack at a 95% confidence level.

d. Risk of Termite Attack

Referring to the analysis that was conducted on the evaluation of the attack frequency and the intensity of the damage to the wood, the level of danger caused by termites at each observation station can be classified into three classes, namely, (1) low, (2) middle, and (3) high, as shown in Table 4.

**Figure 7:**
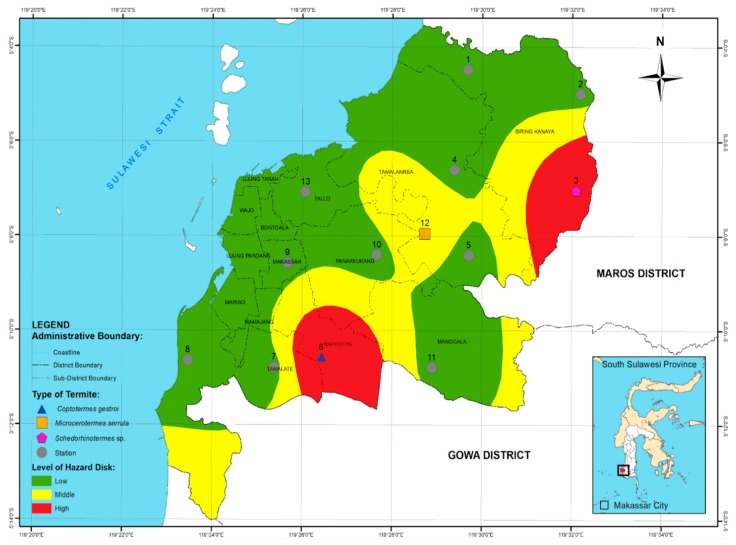
Map of hazard risk of subterranean termite attack in Makassar city, South Sulawesi province.

In Table 4, it can be seen that most areas of Makassar had a low risk (10 stations), although a medium risk of attack was observed at one station and a high risk was found at two stations. Areas shown to have a low risk of subterranean termite attack lie within the following districts: Manggala, Ujung Tanah, Wajo, Tamalate, Rappocini, Makassar, Ujung Pandang, Biring Kanaya, Tamalantea, Mariso, Panakukang, and Tallo. A medium risk of subterranean termite attack was involved parts of Mamajang, Manggala, Ujung Tanah, Wajo, Tamalate, Rappocini, Makassar, Ujung Pandang, Biring Kanaya, Tamalantea, Mariso, Panakukang, and Tallo districts. Meanwhile, the highest attack level was identified in five sub-districts of Makassar City, namely, Manggala, Ujung Tanah, Tamalate, Rappocini, Biring Kanaya, Mariso, and Panakukang. Figure 7 depicts the risk of subterranean termite attack citywide.

## 4. Conclusions

The termite species found in Makassar included *Schedorhinotermes* sp., *C. gestroi*, and *M. serrula*. The environmental factors, consisting of soil characteristics (soil pH, C-organic content, sand, silt, clay, and sand/clay ratio) and climate (temperature and humidity), significantly affected the incidence of subterranean termite attack (p-value = 1.00 < α = 0.05) based on the Wald chi-square test, but they did not affect the intensity and frequency of wood damage arising from subterranean termite attack (p-value = 1.00 < α = 0.05). 

## Figures and Tables

**Table 1:** Classification of wood damage assessment.

Score	Attack Intensity
10	No attack; 1–2% loss from a surface cross section (minor damage)
9	Penetration; 3% from a surface cross section
8	Penetration; 3–10% from a surface cross section
7	Penetration; 10–30% from a surface cross section
6	Penetration; 30–50% from a surface cross section
4	Penetration; 50–75% from a surface cross section
0	Penetration; >75% from a surface cross section

Source: ASTM-D 1758 06 [27].

**Table 2:** Classification of the frequency of termite attacks [28].

Class	Frequency (%)	Description
1	0	Nil (no attack)
2	1–10	Very low
3	11–20	Low
4	21–30	Middle
5	31–40	High
6	>40	Very high

**Table 3:** Risk classification for the danger of termite attacks on the ground.

Intensity	Termite Attack Frequency (%) ^a^					
	0	1–10	11–20	21–30	31–40	>40
10	1	1	1	1	1	2
9	-	1	2	2	2	3
8	-	1	2	2	2	3
7	-	2	2	2	3	3
6	-	2	2	3	3	3
4	-	3	3	3	3	3
0	-	3	3	3	3	3

^a^ Risk classes: 1 = low; 2 = middle; 3 = high.

**Table 4:** Risk class of termite attack in Makassar city.

Code	Low	Middle	High	Termite Species
S1	√	-	-	-
S2	√	-	-	-
S3	-	-	√	*Schedorhinotermes* sp.
S4	√	-	-	-
S5	√	-	-	-
S6	-	-	√	*Coptotermes gestroi*
S7	√	-	-	-
S8	√	-	-	-
S9	√	-	-	-
S10	√	-	-	-
S11	√	-	-	-
S12	-	√	-	*Microcerotermes serrula*
S13	√	-	-	-
